# Persistence of Carbohydrate Availability Shapes Longevity and Reproductive Performance of *Trichogramma chilonis* Ishii (Hymenoptera: Trichogrammatidae)

**DOI:** 10.3390/insects17080852

**Published:** 2026-08-16

**Authors:** Yu Wang, Nan Wang, Zhi-Da Li, Chen Zhang

**Affiliations:** Agricultural College, Jilin Agricultural Science and Technology College, Jilin 132101, China; wangyu@jlnku.edu.cn (Y.W.); wangnan0488@163.com (N.W.); lzz1023080787@gmail.com (Z.-D.L.)

**Keywords:** *Trichogramma chilonis*, adult nutrition, honey–water, sucrose, parasitism, longevity, biological control, mass rearing

## Abstract

This study investigated whether carbohydrate source and the persistence of nutrient availability influence the life-history and reproductive performance of *Trichogramma chilonis*. Seven experimental treatments were evaluated: continuous supply of 20% honey–water (HWC), one-time supply of 20% honey–water with the nutrient source retained throughout the experiment (HW1C), one-time supply of 20% honey–water followed by removal of the nutrient source on Day 2 (HW1), continuous supply of 20% sucrose (SWC), one-time supply of 20% sucrose with the nutrient source retained throughout the experiment (SW1C), one-time supply of 20% sucrose followed by removal of the nutrient source on Day 2 (SW1), and a water-only control (CK). Unlike previous studies that primarily compared carbohydrate types or carbohydrate-fed and unfed parasitoids, the present study specifically assessed the importance of persistent nutrient access. Nutrient-supply treatments significantly increased adult longevity and lifetime parasitism capacity compared with the water-only control. SW1C, SWC, HWC, and HW1C showed statistically comparable longevity and lifetime parasitism capacity and performed better than treatments in which the nutrient source was removed and the water-only control. Daily parasitism was highest during the early adult period and declined with age, although nutrient-fed females maintained parasitism for a longer period. Female progeny ratio varied among experimental treatments, while offspring emergence remained high and statistically similar across the experimental treatments, ranging from approximately 85% to 89%. Overall, honey–water and sucrose diets improved the adult performance of *T. chilonis* without reducing offspring viability, supporting their use in mass-rearing and biological-control programs.

## Simple Summary

*Trichogramma chilonis* Ishii (Hymenoptera: Trichogrammatidae) is an important egg parasitoid of various lepidopteran pests. This study evaluated the effects of carbohydrate-based diets, including honey–water and sucrose solutions, on its survival and reproductive performance. The results showed that sugar-fed parasitoids survived longer and parasitized more host eggs than those provided only with water. The continuous 20% honey–water (HWC), one-time 20% honey–water retained throughout the experiment (HW1C), continuous 20% sucrose (SWC), and one-time 20% sucrose retained throughout the experiment (SW1C) treatments showed similarly strong performances in terms of adult longevity and lifetime parasitism capacity, whereas offspring emergence remained unaffected across treatments. These findings suggest that appropriate carbohydrate supplementation can improve the quality and efficiency of *T. chilonis* for mass rearing and biological pest management programs.

## 1. Introduction

The egg parasitoids of the genus *Trichogramma* (Hymenoptera: Trichogrammatidae) are among the most widely used natural enemies in augmentative biological-control programs [1,2,3]. These minute parasitoids attack the egg stage of numerous lepidopteran pests, thereby preventing larval emergence and reducing subsequent crop damage [4,5,6]. In China, the mass production and release of *Trichogramma* spp. have been implemented on a large scale, particularly for the management of the Asian corn borer in maize. By 2015, approximately 5.5 million ha of maize in northeastern China were protected through *Trichogramma* releases, demonstrating the practical importance of these parasitoids in regional pest-management programs [7,8,9]. The family Trichogrammatidae comprises approximately 620 described species worldwide, of which 219 have been recorded in China [10,11]. Among them, *Trichogramma chilonis* Ishii is an important egg parasitoid of numerous economically important lepidopteran pests [12,13]. It is widely mass-reared and released in China and other Asian countries to control economically important pests of agricultural and horticultural crops [14,15,16,17].

Biological parameters, including longevity, lifetime parasitism capacity, offspring sex ratio, and offspring emergence rate, are critical indicators for evaluating the quality of parasitoid wasps used in biological pest control, and these traits are strongly influenced by adult nutrition [18,19]. Carbohydrates serve as the primary nutritional resource for adult parasitoids, providing the metabolic energy required for essential physiological functions, thereby enhancing key fitness traits [20,21,22,23]. Straser and Wilson [24] revealed that food deprivation significantly reduced the fitness of the egg parasitoid *Hadronotus pennsylvanicus* Ashmead (Hymenoptera: Scelionidae) by decreasing longevity, lifetime fecundity, oviposition activity, and host interaction, demonstrating the importance of carbohydrate nutrition for reproductive performance. Furthermore, Kishani Farahani et al. [25] reported that, in the parasitoid wasp *Venturia canescens* (Gravenhorst) (Hymenoptera: Ichneumonidae), poor-quality diets provided insufficient energy and nutrients to support the metabolically demanding processes of learning, memory formation, and normal brain function, thereby impairing cognitive performance. In natural conditions, parasitoids obtain carbohydrates from nectar, honeydew, and other sugar-rich resources [26], whereas laboratory and mass-rearing systems commonly provide artificial diets such as honey–water or sucrose solutions [27]. In laboratory experiments, Harvey et al. [28] reported that honey significantly improved the fitness of the hyperparasitoids *Lysibia nana* (Gravenhorst) and *Gelis agilis* (Fabricius) (Hymenoptera: Ichneumonidae, Cryptinae) compared with purified sugar solutions, likely because its amino acids, vitamins, minerals, and antioxidants provided physiological benefits beyond carbohydrates alone. Furthermore, Hu et al. [18] also reported that glucose, sucrose, and fructose significantly improved the longevity, parasitism, flight ability, and host-searching behavior of aphid parasitoid, *Binodoxys communis* (Gahan) (Hymenoptera: Aphelinidae), demonstrating the positive role of carbohydrate nutrition in enhancing parasitoid fitness. In augmentative biological control, adult carbohydrate nutrition represents a critical component of mass-rearing protocols because the success of parasitoid releases ultimately depends on the physiological quality and field performance of released individuals [29]. However, previous studies have primarily examined carbohydrate type, while the effect of the duration and persistence of nutrient availability on *T. chilonis* performance remains insufficiently understood. Therefore, the present study investigated not only the effects of carbohydrate sources but also the influence of nutrient-supply regime and persistence of access on the life-history and reproductive performance of *Trichogramma chilonis*. By comparing continuous carbohydrate provision with one-time provision in which the nutrient source was either retained throughout the experiment or removed on Day 2, we specifically assessed whether prolonged nutrient accessibility influences adult longevity, lifetime and daily parasitism, offspring sex ratio, and offspring emergence. This design allowed us to distinguish the effects of persistent carbohydrate availability from those associated solely with carbohydrate identity.

## 2. Materials and Methods

### 2.1. Parasitoids

The adult parasitoids *Trichogramma chilonis* were obtained from parasitized rice stem borer (*Chilo suppressalis* Walker, 1863) (Lepidoptera: Crambidae) eggs collected from rice fields in Changchun, Jilin Province, China (43.89° N, 125.32° E). Based on the morphological characteristics of the male genitalia, the parasitoid species was identified using scanning electron microscope micrographs following Pinto [30], and the identification was further confirmed by rDNA ITS2 sequence analysis according to Stouthamer et al. [31]. In the laboratory, the parasitoids were cultured on rice moth (*Corcyra cephalonica* Stainton, 1866) (Lepidoptera: Pyralidae) eggs for five generations in an incubator (MLR-351H; Sanyo Corporation, Moriguchi, Osaka, Japan) under optimal conditions: L14:D10 photoperiod, 26 ± 1 °C, and 65 ± 5% RH. They were then maintained on their native host, *C. suppressalis* eggs, for one generation. Newly emerged (<12 h), fully mated female wasps were used in the experiments.

### 2.2. Rearing of Rice Moth, Corcyra cephalonica (Stainton, 1866)

To obtain host eggs of the *C. cephalonica* for rearing *Trichogramma parasitoids*, *C. cephalonica* larvae were maintained in a climate chamber (MLR-351H; Sanyo Corporation, Moriguchi, Osaka, Japan) under controlled conditions of 25 ± 1 °C, 70 ± 5% RH, and a 14:10 h light:dark photoperiod. The larvae were fed an artificial diet consisting of 500 g corn flour, 142 g wheat bran, 21 g yeast, 49 g sucrose, and 200 mL distilled water. This cereal-based formulation was selected because comparable diets containing approximately 70% corn flour, 20% wheat bran, 7% sugar, and 3% yeast have been successfully used for the laboratory rearing of *C. cephalonica* [32]. Larvae were reared in polypropylene (PP) plastic containers (23 cm × 23 cm × 5 cm; Bugdorm-I, Taiwan, China). After pupation, the pupae were collected and transferred to mesh cages (35 cm × 22 cm × 42 cm; Bugdorm-I, Taiwan, China) for adult emergence and oviposition. Eggs laid by adult moths were collected on stainless steel trays (35 cm × 22 cm × 42 cm; Bosom Metal Co., Ltd., Foshan, China), and impurities, including moth scales, were removed using a 0.50-mm-aperture stainless-steel woven-wire sieve, 200 mm in diameter (RETSCH GmbH, Haan, Germany). Fresh eggs laid within 24 h were collected daily and exposed for 30 min to UV-C radiation from a 30-W germicidal lamp emitting at 254 nm (G30T8, Sankyo Denki, Hiratsuka, Japan), positioned approximately 15 cm above the eggs in an enclosed chamber. This treatment was used to arrest embryonic development before parasitization.

### 2.3. Effects of Nutrient Supply on Life-History Traits and Reproductive Performance

**Figure 1:**
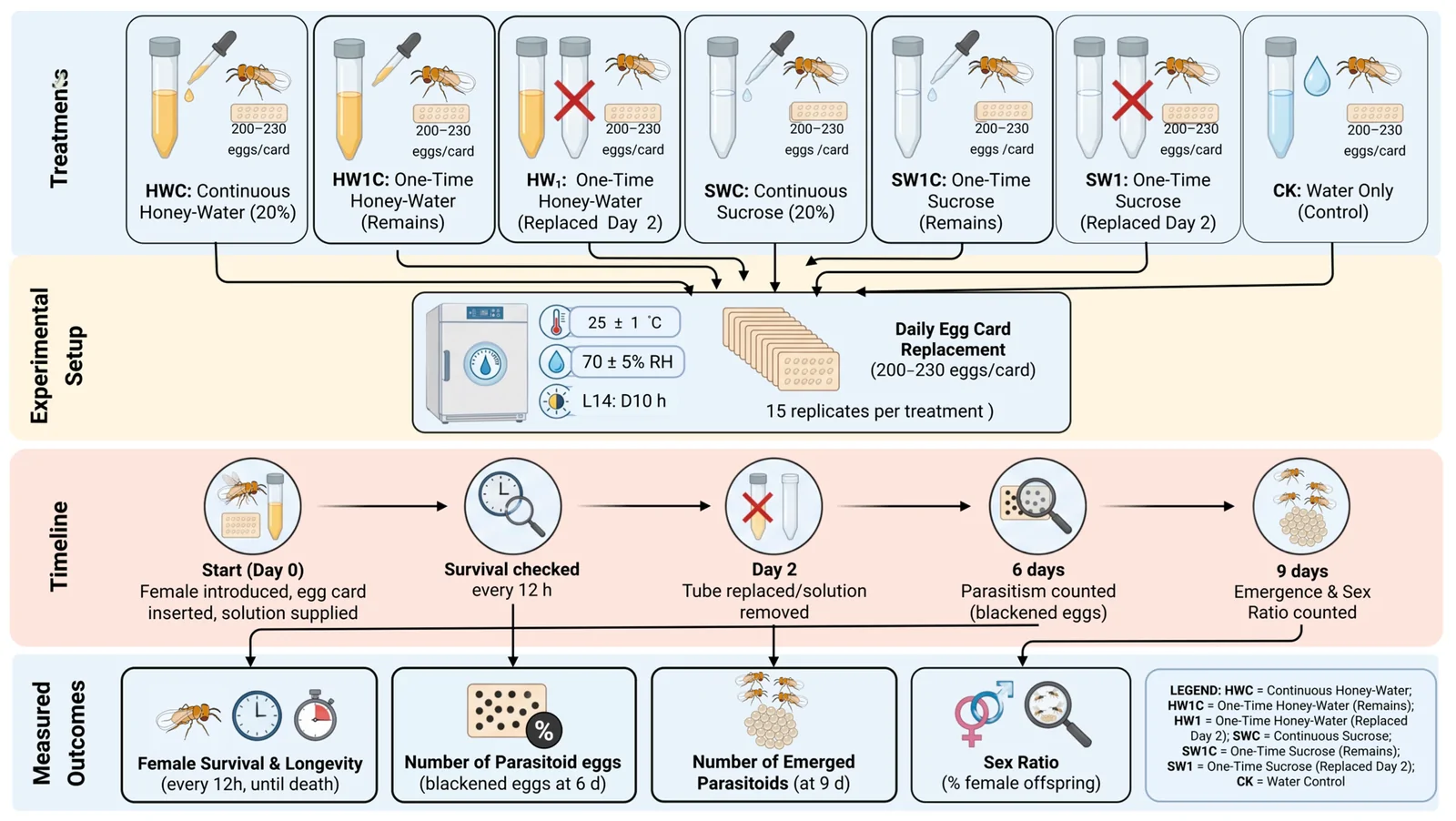
Schematic overview of the experimental design, timeline, and measured outcomes in female *Trichogramma Chilonis*. Created in BioRender. Zhang, C. (2026) https://BioRender.com/ez5am7a (accessed on 13 August 2026).

To evaluate the effects of nutrient supply on the biological performance of *Trichogramma chilonis*, one newly emerged and fully mated female was individually placed in a glass tube (10 cm × 3 cm; Xinxiang Qianxuan Glass Products Co., Ltd., Jiaozuo, China). Each tube contained an egg card, consisting of a small rectangular piece of white cardboard (approximately 0.5 cm × 1 cm), onto which 200–230 freshly UV-irradiated *C. cephalonica* eggs were evenly distributed and securely affixed using a thin layer of non-toxic, water-soluble adhesive (diluted gum arabic solution). Honey and sucrose were selected as low-cost, readily available carbohydrate sources that are commonly used in laboratory and mass-rearing systems for parasitoids. Seven experimental treatments were established, comprising six nutrient-supply treatments and one water control (CK). The six nutrient-supply treatments consisted of a continuous supply of 20% honey–water solution (HWC), a one-time supply of 20% honey–water solution with the solution remaining in the tube (HW1C), a one-time supply of 20% honey–water solution followed by replacement with a clean tube on day 2 (HW1), a continuous supply of 20% sucrose solution (SWC), a one-time supply of 20% sucrose solution with the solution remaining in the tube (SW1C), and a one-time supply of 20% sucrose solution followed by replacement with a clean tube on day 2 (SW1). The host egg card was replaced daily with a fresh card containing 200–230 eggs. Each removed egg card was labelled according to experimental treatment and replicate and was transferred to a new tube under the same controlled conditions of 25 ± 1 °C, 70 ± 5% relative humidity, and a L14:D10 h photoperiod. Female survival was observed every 12 h, and longevity was recorded in half-day units until death. Six days after parasitism, the number of blackened host eggs was counted to determine the level of parasitism. After 9 days, the number of emerged parasitoids was recorded, and the sex ratio was calculated as the proportion of female offspring among the total emerged adults. Each experimental treatment was initially established with 15 biological replicates (Figure 1).

### 2.4. Statistical Analysis

Longevity, lifetime parasitism capacity, female progeny proportion, and offspring emergence rate were analyzed separately using one-way ANOVA in IBM SPSS Statistics 28.0, with nutrient-supply treatment as the fixed factor. When significant treatment effects were detected, means were compared using Tukey’s HSD test at *p* < 0.05. Partial eta squared (ηp^2^) was calculated as an effect-size measure to quantify the magnitude of treatment effects. Percentage data, including female progeny proportion and offspring emergence rate, were arcsine square-root-transformed before analysis, whereas untransformed means ± SE are presented for interpretation.

Daily parasitism was analyzed using a generalized linear mixed-effects model (GLMM) in Python 3.13.5 using the statsmodels 0.14.6 statistical package. A Poisson distribution with a log-link function was specified because parasitism was recorded as count data. Treatment, day, and their interaction (Treatment × Day) were included as fixed effects, whereas female identity was included as a random intercept to account for repeated observations and within-female correlation. Day was treated as a categorical factor, and fixed effects were evaluated using likelihood ratio tests. Data are graphically presented using SigmaPlot 12.5.

## 3. Results

### 3.1. Effect of Nutrient Supply on Trichogramma chilonis Longevity

**Figure 2:**
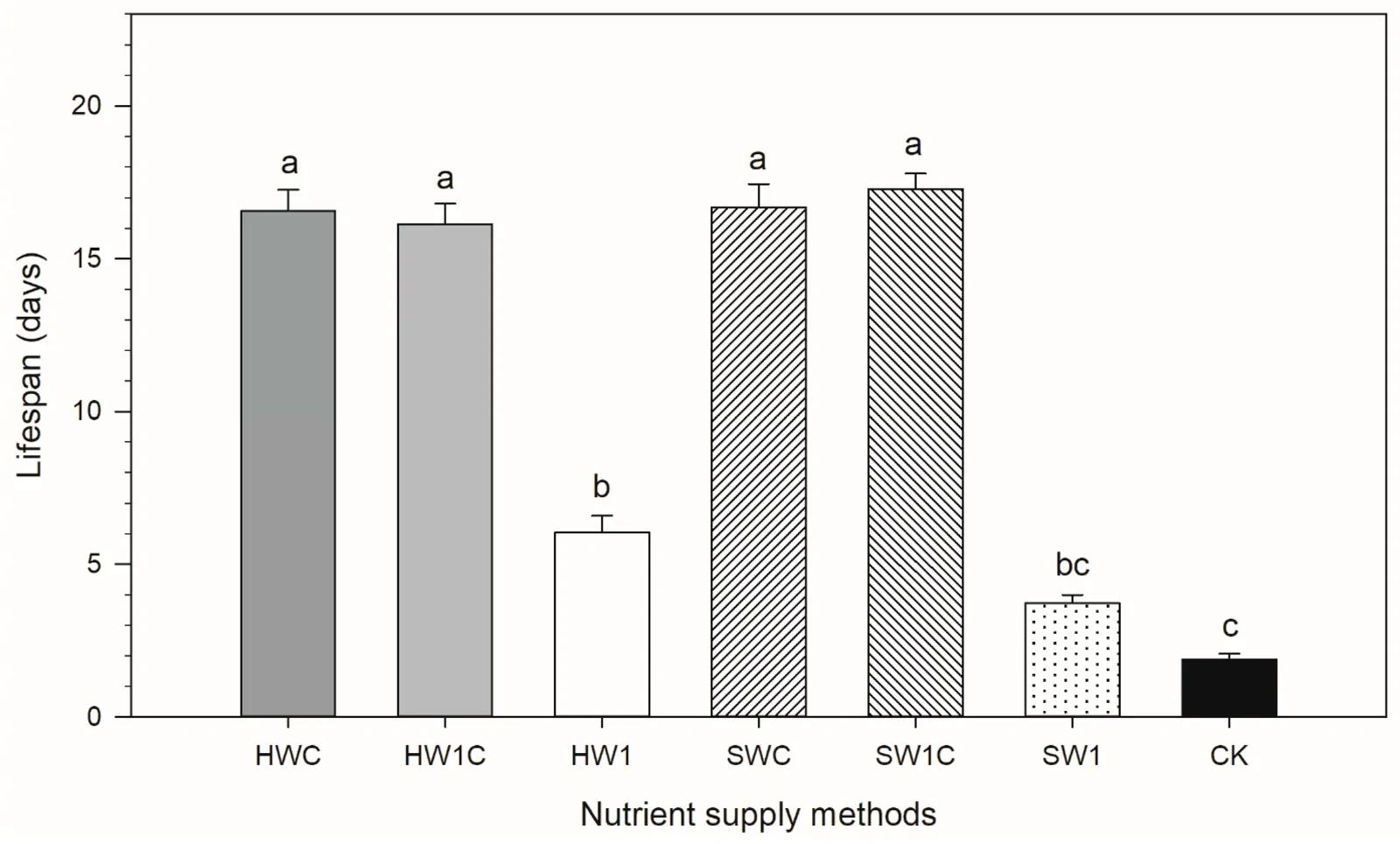
Mean longevity (days ± SE) of *Trichogramma chilonis* adults under different experimental treatments. HWC represents the continuous supply of 20% honey–water solution; HW1C indicates a one-time supply of 20% honey–water solution, with the solution retained in the tube throughout the experiment; HW1 denotes a one-time supply of 20% honey–water solution, followed by replacement with a clean tube on day 2. SWC represents the continuous supply of 20% sucrose solution; SW1C indicates a one-time supply of 20% sucrose solution, with the solution retained in the tube throughout the experiment; SW1 denotes a one-time supply of 20% sucrose solution, followed by replacement with a clean tube on day 2. CK represents the water-only control. Different lowercase letters above the bars indicate significant differences among treatments, whereas bars sharing the same letter are not significantly different (Tukey’s HSD test, *p* < 0.05).

The longevity of *T. chilonis* differed significantly among the experimental treatments (F_6,90_ = 151.507, *p* < 0.001, ηp^2^ = 0.910), indicating a very large treatment effect. SW1C, SWC, HWC, and HW1C showed statistically similar longevity, with mean values ranging from 16.1 to 17.3 days. In contrast, longevity was substantially lower in HW1 (6.0 days), SW1 (3.7 days), and CK (1.9 days). Relative to CK, SW1C extended mean adult longevity by 15.4 days, corresponding to approximately a 9.1-fold difference. However, SW1C was not statistically superior to HWC, HW1C, or SWC (Figure 2).

### 3.2. Effect of Nutrient Supply on Lifetime Parasitism Capacity of Trichogramma chilonis

**Figure 3:**
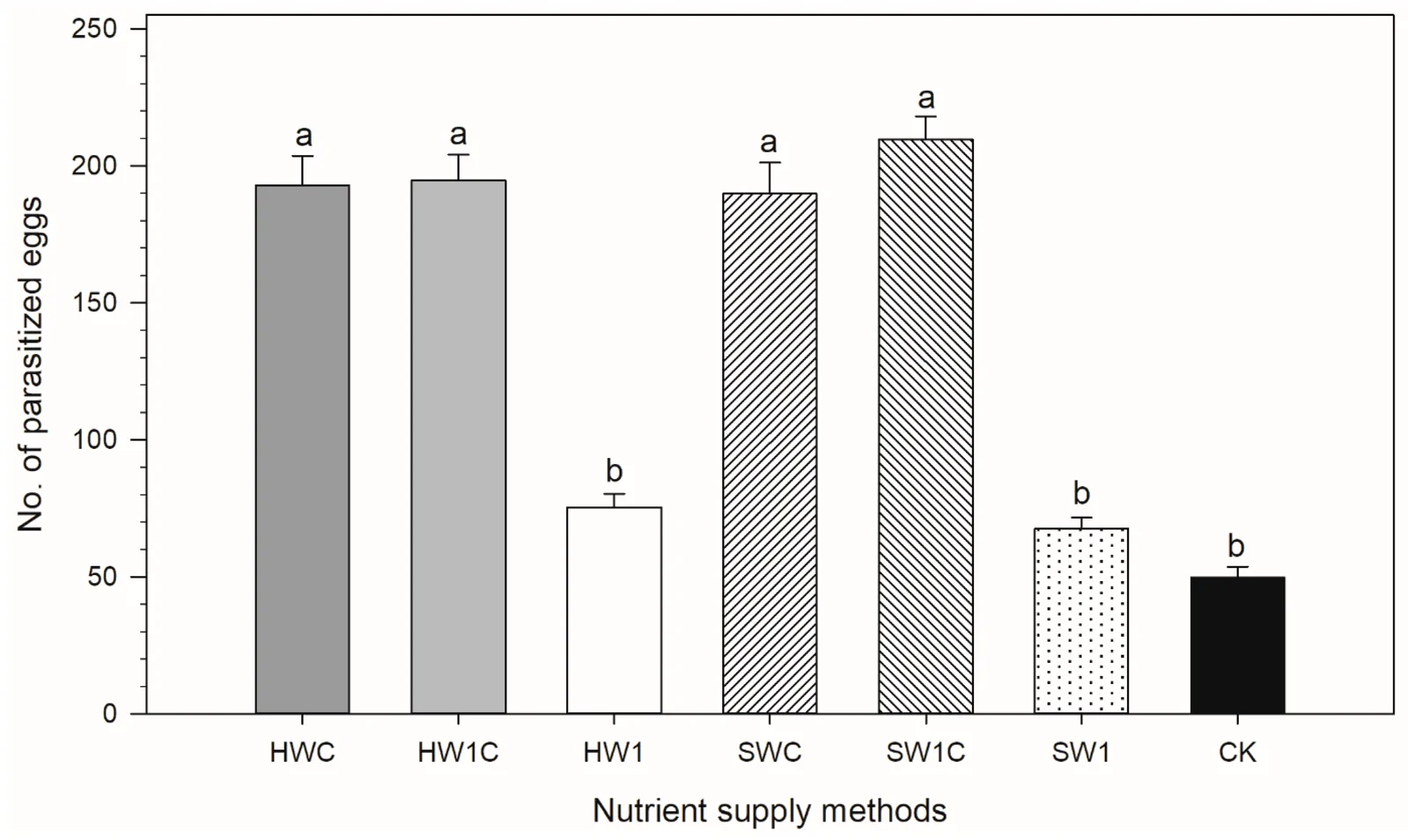
Mean lifetime parasitism capacity (number of eggs parasitized ± SE) of *Trichogramma chilonis* under different experimental treatments. HWC represents the continuous supply of 20% honey–water solution; HW1C indicates a one-time supply of 20% honey–water solution, with the solution retained in the tube throughout the experiment; HW1 denotes a one-time supply of 20% honey–water solution, followed by replacement with a clean tube on day 2. SWC represents the continuous supply of 20% sucrose solution; SW1C indicates a one-time supply of 20% sucrose solution, with the solution retained in the tube throughout the experiment; SW1 denotes a one-time supply of 20% sucrose solution, followed by replacement with a clean tube on day 2; and CK represents the water-only control. Different lowercase letters above the bars indicate significant differences among treatments, whereas bars sharing the same letter are not significantly different (Tukey’s HSD test, *p* < 0.05).

Lifetime parasitism capacity differed significantly among the experimental treatments (F_6,90_ = 73.730, *p* < 0.001, ηp^2^ = 0.831), demonstrating a very large treatment effect. Lifetime parasitism capacity was statistically similar among SW1C, HW1C, HWC, and SWC, with mean values ranging from 189.9 to 209.7 for parasitized eggs. In contrast, lifetime parasitism capacity was significantly lower in HW1 (75.3 eggs), SW1 (67.5 eggs), and CK (49.8 eggs). Compared with CK, SW1C produced 159.9 additional parasitized eggs, equivalent to an approximately 4.2-fold difference (Figure 3).

### 3.3. Effect of Nutrient Supply on Daily Parasitism of Trichogramma chilonis

Daily parasitism was significantly influenced by nutrient-supply treatment and adult age. The Poisson generalized linear mixed-effects model revealed a significant overall effect of treatment (likelihood ratio χ^2^ = 89.96, df = 6, *p* < 0.001) and a pronounced effect of day (likelihood ratio χ^2^ = 3905.29, df = 6, *p* < 0.001). Importantly, the Treatment × Day interaction was also significant (likelihood ratio χ^2^ = 270.56, df = 32, *p* < 0.001), demonstrating that temporal changes in parasitism differed among nutrient-supply treatments.

**Figure 4:**
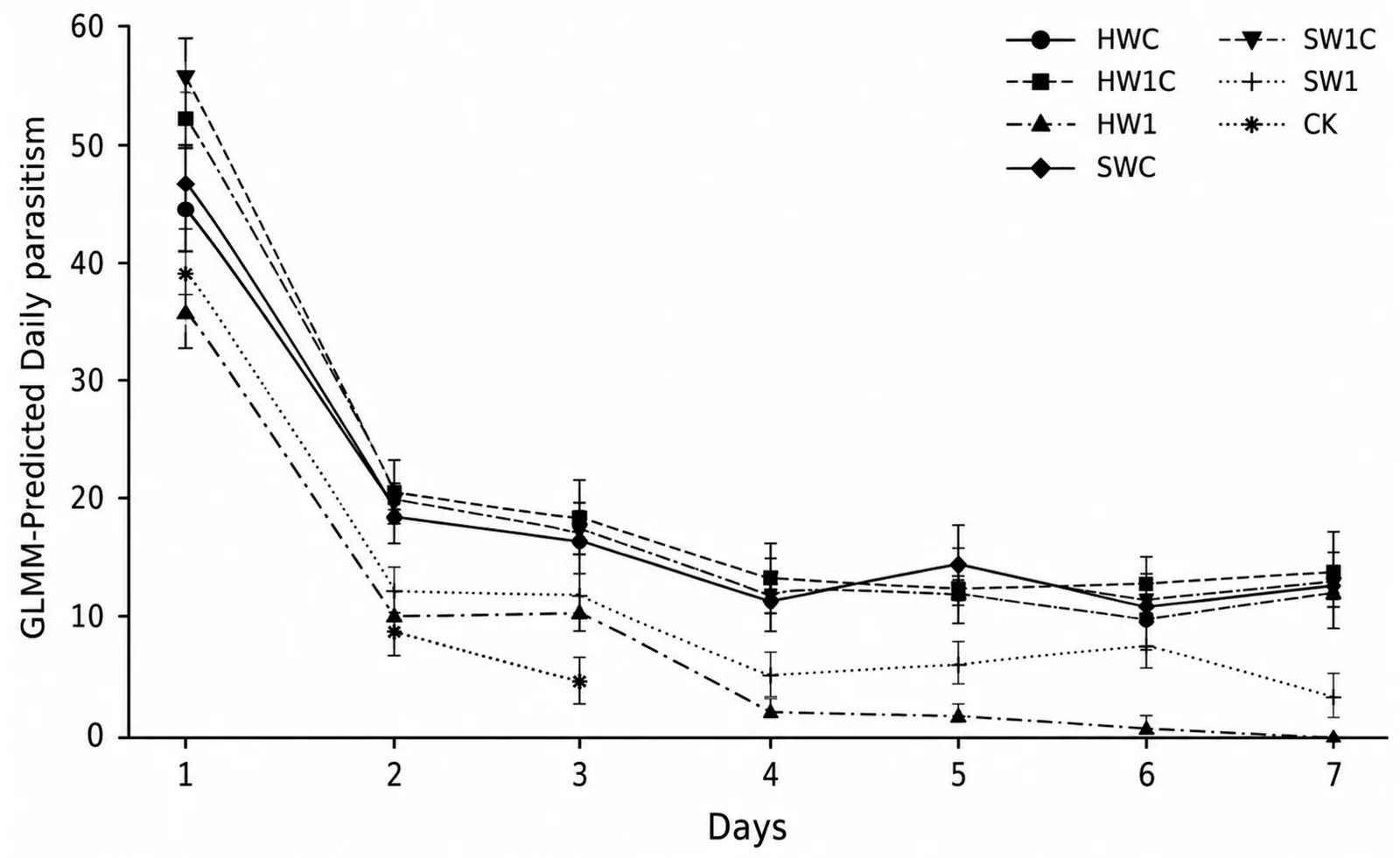
Model-predicted daily parasitism of *Trichogramma chilonis* under different nutrient-supply treatments based on a Poisson generalized linear mixed-effects model (GLMM). Points represent predicted means and error bars indicate 95% confidence intervals. HWC, continuous 20% honey–water; HW1C, one-time honey–water retained throughout the experiment; HW1, one-time honey–water removed on Day 2; SWC, continuous 20% sucrose; SW1C, one-time sucrose retained throughout the experiment; SW1, one-time sucrose removed on Day 2; CK, water-only control.

Model-predicted daily parasitism was highest during the first day of adult life. On Day 1, predicted parasitism ranged from approximately 35.76 eggs per female in HW1 to 54.49 eggs per female in SW1C. Predicted values were 46.40, 51.76, 35.76, 45.50, 54.49, 38.72, and 40.60 eggs per female for HWC, HW1C, HW1, SWC, SW1C, SW1, and CK, respectively. Parasitism declined sharply after Day 1 in all treatments and subsequently followed treatment-specific trajectories (Figure 4).

Females supplied continuously with carbohydrate or provided with a one-time nutrient source that remained accessible showed comparatively sustained reproductive activity during later observation days. By Day 7, model-predicted daily parasitism remained at 13.20 eggs in HWC, 13.79 eggs in HW1C, 12.12 eggs in SWC, and 12.29 eggs in SW1C. In contrast, removal of the nutrient source resulted in substantially greater late-life declines. Predicted parasitism in HW1 decreased to 3.26 eggs per female by Day 7, whereas SW1 declined from 38.72 eggs on Day 1 to approximately zero by Day 7.

The water-only control also showed a rapid decline, with predicted parasitism decreasing from 40.60 eggs per female on Day 1 to 9.29 on Day 2 and 5.27 on Day 3. No CK daily parasitism observations were available after Day 3 because females did not survive to provide subsequent daily measurements. Overall, the significant Treatment × Day interaction indicates that persistent carbohydrate availability not only prolonged reproductive activity but also modified the temporal pattern of daily parasitism (Figure 4).

### 3.4. Effect of Nutrient Supply on Offspring Female Ratio of Trichogramma chilonis

**Figure 5:**
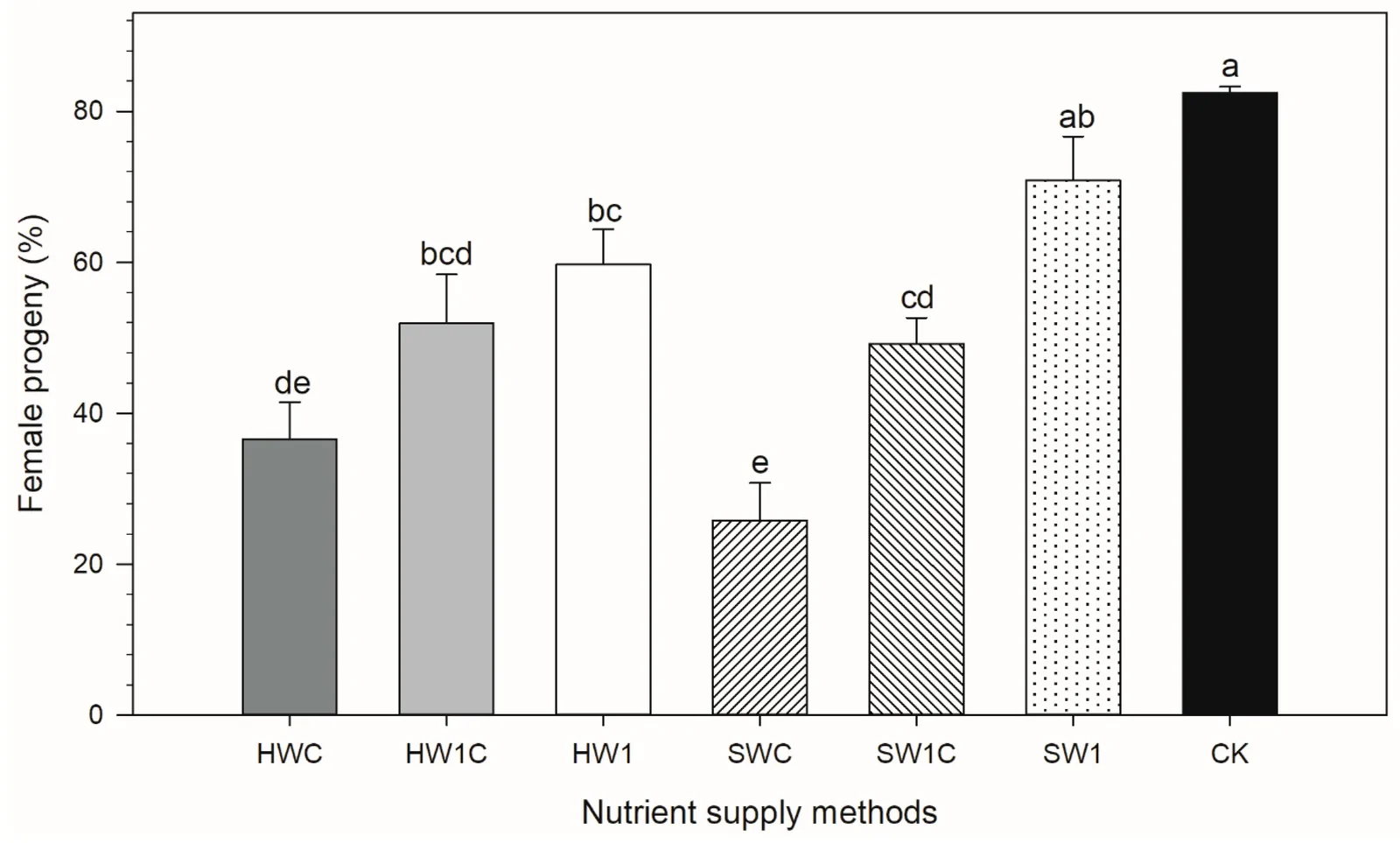
Mean percentage of female progeny (±SE) of *Trichogramma chilonis* under different experimental treatments. HWC represents the continuous supply of 20% honey–water solution; HW1C indicates a one-time supply of 20% honey–water solution, with the solution retained in the tube throughout the experiment; HW1 denotes a one-time supply of 20% honey–water solution, followed by replacement with a clean tube on day 2. SWC represents the continuous supply of 20% sucrose solution; SW1C indicates a one-time supply of 20% sucrose solution, with the solution retained in the tube throughout the experiment; SW1 denotes a one-time supply of 20% sucrose solution, followed by replacement with a clean tube on day 2; and CK represents the water-only control. Different lowercase letters above the bars indicate significant differences among treatments, whereas bars sharing the same letter are not significantly different (Tukey’s HSD test, *p* < 0.05).

Female progeny proportion differed significantly among the experimental treatments (F_6,90_ = 16.846, *p* < 0.001, ηp^2^ = 0.529), indicating a large treatment effect. CK produced the highest female progeny proportion (82.4%), whereas SWC produced the lowest female-biased offspring production (25.8%), representing an absolute difference of 56.6 percentage points. Intermediate values were recorded for SW1 (70.8%), HW1 (59.7%), HW1C (51.9%), SW1C (49.2%), and HWC (36.6%) (Figure 5).

### 3.5. Effect of Nutrient Supply on Offspring Emergence Rate of Trichogramma chilonis

**Figure 6:**
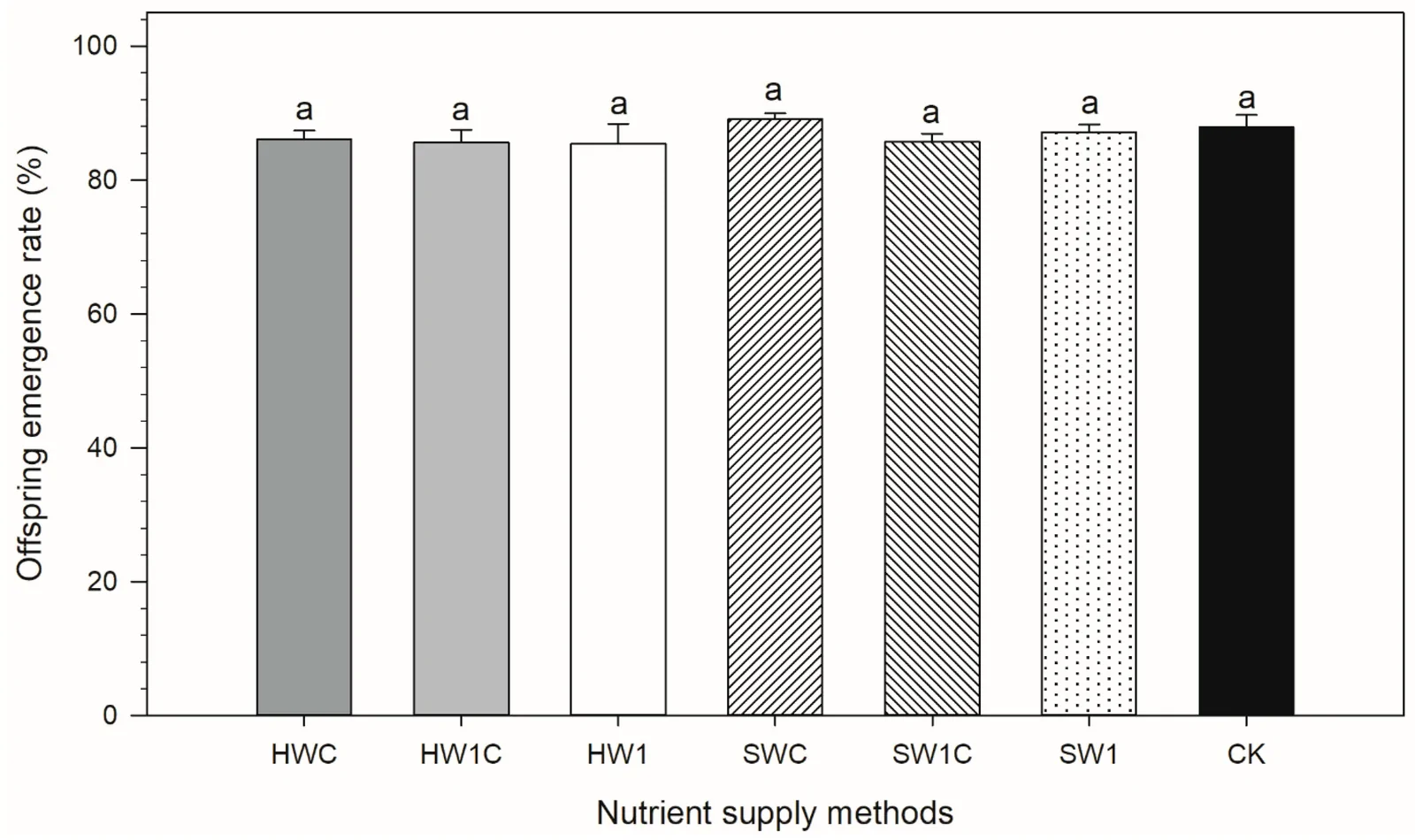
Mean percentage of offspring emergence (±SE) of *Trichogramma chilonis* under different experimental treatments. HWC represents the continuous supply of 20% honey–water solution; HW1C indicates a one-time supply of 20% honey–water solution, with the solution retained in the tube throughout the experiment; HW1 denotes a one-time supply of 20% honey–water solution, followed by replacement with a clean tube on day 2. SWC represents the continuous supply of 20% sucrose solution; SW1C indicates a one-time supply of 20% sucrose solution, with the solution retained in the tube throughout the experiment; SW1 denotes a one-time supply of 20% sucrose solution, followed by replacement with a clean tube on day 2; and CK represents the water-only control. The same lowercase letter above the bars indicates that offspring emergence did not differ significantly among treatments (Tukey’s HSD test, *p* > 0.05).

The offspring emergence rate of *T. chilonis* did not differ significantly among experimental treatments (F_6,90_ = 0.640, *p* = 0.698, ηp^2^ = 0.041). The small effect size and narrow observed range of approximately 85–89% further indicate that the experimental treatments had limited influence on offspring emergence (Figure 6).

## 4. Discussion

The longevity results observed in the present study are consistent with the well-established beneficial effects of carbohydrate feeding in *Trichogramma*; however, our findings extend this knowledge by highlighting the importance of the pattern and persistence of nutrient availability. Wang et al. [19], for example, primarily compared carbohydrate-fed and water-fed treatments across four *Trichogramma* species and focused on egg load and population parameters, whereas Tian et al. [26] evaluated the physiological benefits of different naturally occurring sugars in *Trichogramma chilonis* and *T. japonicum*. Similarly, Leatemia et al. [33] compared several adult food sources in *T. minutum* to determine their effects on longevity, fecundity, and offspring sex ratio. Adult body size may represent another source of variation in longevity among studies. Grenier et al. [34] demonstrated that the body size of *Trichogramma* adults can vary according to host species and the number of parasitoids developing within a host egg, indicating that differences in host resource availability may influence adult phenotype. Under mass-rearing conditions, variation in host utilization and the number of developing parasitoids per host may therefore contribute to differences in adult body size and potentially to variation in fitness-related traits, including longevity. Because adult body size was not measured in the present study, its possible contribution to the observed variation in longevity cannot be directly assessed. In contrast, the present experiment compared continuous nutrient provision with a one-time provision in which the food remained available in the experimental tube and a one-time provision followed by removal of the food source on Day 2. This distinction revealed that the duration of nutrient accessibility was at least as important as carbohydrate identity. Females in SW1C, SWC, HWC, and HW1C survived for 16.1–17.3 days, whereas longevity declined markedly to 6.0 and 3.7 days in HW1 and SW1, respectively, after the nutrient source was removed, and to only 1.9 days in the water-only control. Notably, longevity did not differ significantly among SW1C, SWC, HWC, and HW1C, with mean values ranging from 16.1 to 17.3 days. The comparable performance of the retained one-time treatment (SW1C) and the continuously supplied treatments suggests that repeated replenishment may not be essential when a suitable carbohydrate source remains accessible. Thus, rather than simply confirming that carbohydrates prolong *T. chilonis* survival, our results extend previous findings by demonstrating that the persistence of access to the carbohydrate source is a key determinant of adult longevity. This distinction may have practical implications for mass-rearing systems, where reducing the frequency of diet replacement while maintaining nutrient accessibility could simplify parasitoid maintenance.

Lifetime parasitism capacity showed a pattern broadly consistent with adult longevity. *Trichogramma chilonis* females assigned to the nutrient-supply treatments, particularly SW1C, HW1C, HWC, and SWC, parasitized the highest number of host eggs. In contrast, markedly lower parasitism was recorded in HW1, SW1, and CK. This means that the reproductive benefit of nutrient supply was not limited to adult survival; it also supported host-searching and oviposition activity. Females that lived longer had more time and energy to locate and parasitize host eggs. This interpretation is supported by Wang et al. [19], who found that carbohydrate feeding increased fecundity and improved population parameters in *T. chilonis*. Similarly, Tuncbilek et al. [35] demonstrated that the type of honey provided to *Trichogramma* females significantly influenced both parasitism and longevity, indicating that not only carbohydrate availability but also food source composition can affect parasitoid performance.

Among the nutrient-supply treatments, the strong performance of the sucrose-based treatments was particularly notable. This result is consistent with Tian et al. [26], who demonstrated that sucrose and several other naturally occurring sugars prolonged longevity and increased fecundity in *T. chilonis*. At the mechanistic level, Liu et al. [36] identified gustatory receptors involved in sucrose detection in *T. chilonis*, confirming that this parasitoid possesses a specific sensory mechanism for recognizing sucrose. Together, these findings provide both physiological and sensory support for the beneficial response to sucrose observed in the present study.

The honey–water treatments also performed well, especially HWC and HW1C. Honey is a complex carbohydrate source containing sugars and small quantities of other nutritional components. Its beneficial effects on *Trichogramma* performance have been demonstrated previously. Leatemia et al. [33] reported substantial increases in longevity and offspring production in honey-fed *T. minutum*, while Tuncbilek et al. [35] showed that different honey sources influenced longevity and parasitism in *Trichogramma* parasitoids. Earlier evidence also supports the beneficial role of honey-based adult nutrition in *Trichogramma*. Hohmann and Oatman [37] reported increased longevity in adult *T. platneri* when honey was available, regardless of host presence. Similarly, Mashal et al. [38] evaluated honey and other honeybee products, their mixtures, and sugar solution in three *Trichogramma* species and found that honey-containing diets generally improved adult longevity and fecundity, whereas emergence was unaffected by most dietary treatments. These findings further indicate that honey-based supplementation can enhance adult parasitoid performance, although the magnitude of the response may vary with parasitoid species and diet composition.

Similar nutritional benefits of honey and other sugar-rich diets have also been reported in other parasitoid taxa [28,39]. The high longevity and parasitism observed under HWC and HW1C in the present study are therefore consistent with the broader evidence that access to suitable carbohydrate sources enhances parasitoid performance.

The female offspring proportion responded differently from longevity and lifetime parasitism capacity. The CK produced the highest proportion of female offspring, whereas SWC produced the lowest value. Because *T. chilonis* is arrhenotokous, fertilized eggs develop into females, whereas unfertilized eggs develop into males [40,41]. Differences in adult female longevity among treatments may therefore have influenced the availability of stored sperm and, consequently, offspring sex allocation. Females that survived longer under sustained carbohydrate access may have experienced a greater number of oviposition events, potentially leading to the progressive depletion of sperm stored in the spermatheca. Sustained carbohydrate access can substantially increase longevity and reproductive performance in *T. chilonis* and other *Trichogramma* species [26,33]. In *T. minutum*, long-lived, honey-fed females produced increasingly male-biased broods and eventually exclusively male offspring late in life, a pattern consistent with the progressive depletion of stored sperm [33,42]. Therefore, the lower female offspring proportion observed under sustained carbohydrate access in the present study may reflect prolonged reproductive activity, greater cumulative parasitism, and a progressive reduction in sperm availability. Food and host availability may also influence egg maturation, host acceptance, and reproductive output in *T. chilonis* and related *Trichogramma* species, thereby indirectly affecting sex allocation [43]. However, because sperm reserves, oviposition sequence, and age-specific offspring sex ratios were not measured directly, these mechanisms remain hypothetical. Importantly, a high female proportion alone does not necessarily indicate superior biological-control performance. Although CK produced the highest female proportion, it also showed the shortest longevity and low lifetime parasitism capacity. Therefore, the biological quality of *T. chilonis* should be evaluated by considering longevity, lifetime and daily parasitism, offspring sex ratio, and emergence together rather than sex ratio alone.

Offspring emergence remained high and statistically similar across all experimental treatments, ranging from approximately 85% to 89%, indicating that the effects of nutrient supply on adult longevity and parasitism were not accompanied by detectable differences in successful offspring emergence. A comparable response was reported by Yan et al. [44] for *Trichogramma pintoi*, in which emergence remained statistically similar among 10% sucrose, glucose, honey, and water treatments (93.47–94.74%), although complete starvation significantly reduced emergence to 84.60%. In contrast, Bari et al. [45] reported significantly greater emergence in *Trichogramma zahiri* supplied with 25% honey solution together with host food than in the water-fed control. These studies differed substantially in experimental design: Yan et al. [44] used *T. pintoi* on *Heortia vitessoides* eggs with 10% carbohydrate solutions, whereas Bari et al. [45] used *T. zahiri* on *Dicladispa armigera* eggs with 25% dietary supplements. The present study, however, evaluated *T. chilonis* on *Corcyra cephalonica* eggs using 20% honey–water and sucrose solutions under different nutrient-availability regimes. Thus, the contrasting emergence responses may reflect differences in parasitoid and host species, diet formulation and concentration, and feeding regime, although these factors were not directly tested as the causes of the discrepancies. Although the present study quantified adult female longevity, daily and lifetime parasitism capacity, offspring sex ratio, and emergence, the experimental design did not follow individual offspring through successive developmental stages or record sex-specific adult longevity. Consequently, a complete age-stage, two-sex life table analysis could not be performed, and future studies incorporating developmental duration, stage-specific survival, and reproductive data for both sexes would provide a more comprehensive assessment of population-level responses to nutrient availability.

Overall, HWC, HW1C, SWC, and SW1C formed a statistically comparable group with high longevity and lifetime parasitism capacity. From an applied biological-control perspective, these nutrient-supply treatments may improve parasitoid quality by extending adult survival and increasing lifetime parasitism capacity, while having no significant effect on offspring emergence. However, because the female progeny ratio was higher in CK and SW1, the final treatment recommendation should not be based on female ratio alone. The practical value of a treatment should therefore be evaluated by considering adult survival, lifetime parasitism capacity, female offspring production, and the absence of detectable adverse effects on offspring emergence.

## 5. Conclusions

This study demonstrates that the biological response of *Trichogramma chilonis* to adult carbohydrate nutrition depends not only on carbohydrate source but also on the persistence of nutrient availability. Continuous access and one-time provision with the nutrient source retained throughout the experiment produced similarly high longevity and lifetime parasitism capacity, whereas removal of the nutrient source on Day 2 markedly reduced both traits. These findings suggest that repeated replenishment may not be essential when a suitable carbohydrate source remains accessible. Experimental treatments also influenced offspring sex allocation, whereas offspring emergence remained consistently high and statistically unaffected. Collectively, these results extend previous evidence on the benefits of carbohydrate feeding by identifying the persistence of nutrient access as an important component influencing *T. chilonis* performance. The findings may therefore contribute to the development of simpler and more efficient feeding protocols for laboratory maintenance and mass-rearing programs.

## Data Availability

The raw data supporting the conclusions of this article will be made available by the authors on request.
